# Advancing ^19^F NMR Prediction of Metal-Fluoride Complexes in Solution: Insights from Ab Initio Molecular Dynamics

**DOI:** 10.1021/acs.jpca.4c05408

**Published:** 2024-12-03

**Authors:** Sahil Gahlawat, Kathrin H. Hopmann, Abril C. Castro

**Affiliations:** † Department of Chemistry, UiT The Arctic University of Norway, 9037 Tromsø, Norway; ‡ Hylleraas Centre for Quantum Molecular Sciences, Department of Chemistry, UiT The Arctic University of Norway, 9037 Tromsø, Norway; § Hylleraas Centre for Quantum Molecular Sciences, Department of Chemistry, University of Oslo, 0315 Oslo, Norway

## Abstract

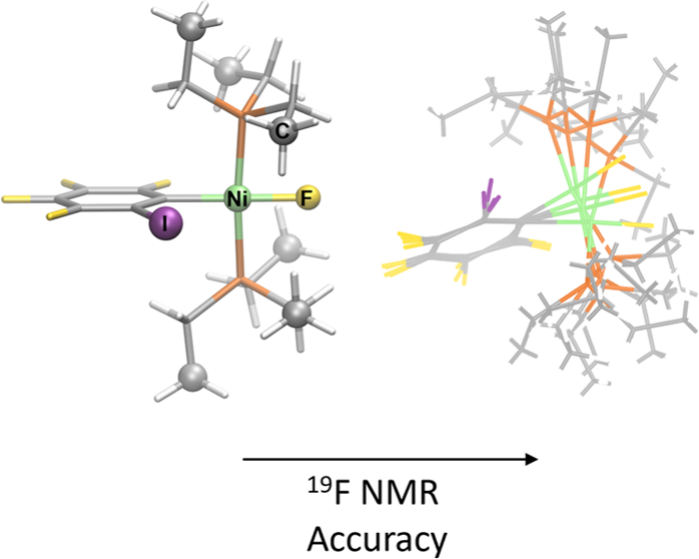

^19^F NMR parameters are versatile probes for studying metal-fluoride complexes. Quantum chemical calculations of ^19^F NMR chemical shifts enhance the accuracy and validity of the resonance signal assignments in complex spectra. However, the treatment of solvation effects in these calculations remains challenging. In this study, we establish a successful computational protocol using ab initio molecular dynamics simulations for the accurate prediction of ^19^F NMR chemical shifts in square-planar nickel-fluoride complexes. In particular, we have studied in detail the *trans*-[NiF(2,3,4,5-C_6_F_4_I)(PEt_3_)_2_] complex in a benzene solution. Our computations revealed that accounting for the dynamic conformational flexibility of the complex, including intramolecular interactions, is crucial for obtaining reliable ^19^F NMR chemical shifts. Overall, this study advances the understanding of employing state-of-the-art quantum chemistry methods to accurately model ^19^F NMR chemical shifts of nickel-fluoride complexes, emphasizing the importance of addressing solvation effects in such calculations.

## 1 Introduction

Metal-fluoride complexes have gained significant interest owing to their unique and interesting catalytic properties, which are of high importance in the pharmaceutical, agrochemical, and advanced materials industries.^1,2^ Their reactivity patterns are remarkably different from those of their more well-known alkoxy, chloro, bromo, and iodo counterparts, influenced by the distinctive properties of fluorine.^3,4^ Fluoride’s tendency to form stronger bonds with early transition metals or metals in high oxidation states is primarily attributed to its small size and high electronegativity. It can also act as a π-donor, enhancing activation by both the metal and the fluoride. In addition, fluoride’s ability to form strong hydrogen bonds and halogen bonds can facilitate further coordination of substrates near the metal atom, enhancing both the stability and reactivity of metal-fluoride complexes.^5^

Nuclear magnetic resonance (NMR) parameters of fluorine serve as highly versatile experimental probes for the molecular structure and chemical bonding of metal-fluoride complexes.^6−8^ The spin-1/2 ^19^F nucleus, being 100% naturally abundant, exhibits a NMR span range of ∼1300 ppm^9^ in general and ∼300 ppm for organofluoride compounds.^10,11^ Moreover, metal-fluorides are particularly intriguing because they exhibit a ^19^F NMR resonance that lies upfield of most signals derived from carbon-bound fluorine. However, the sensitivity of ^19^F NMR shifts to the chemical environment means that even slight variations in the metal’s coordination sphere can significantly change the observed spectra, complicating the assignment of the resonance signals.^12^ The effectiveness and accuracy of ^19^F NMR analysis can therefore be enhanced by theoretical calculations, particularly when the spectra exhibit multiple resonances that are difficult to interpret in a straightforward manner.

Overall, the accuracy of ^19^F NMR chemical shift calculations is influenced by many factors, including the level of theory, geometry optimization, rovibrational corrections, and relativistic effects.^9,13−16^ However, few studies have addressed the influence of the solvation effects. Recent investigations confirmed that specific solute–solvent interactions significantly impact the ^19^F NMR shifts of fluoride-type anions, where fluoride exhibits strong hydrogen bonding interactions with the CH bonds of organic solvents.^17,18^ Notably, these strong interactions are not covered by implicit solvent models and become evident only with explicit solvation treatment.

**Figure 1:**
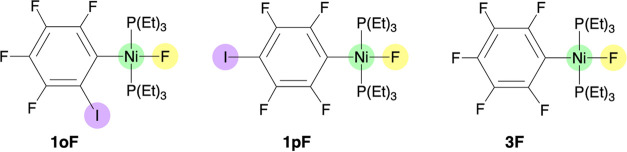
Nickel-fluoride complexes were examined in this work. The labels used in refs (21) and (22) are kept for easier connection with this work.

Since ^19^F NMR is sensitive to halogen bonding interactions, it has proven to be particularly useful in detecting these types of interactions, both in solution^19^ and in the solid state.^20^ By detecting and studying halogen bonding, we gain valuable insights into molecular interactions and the formation of stable structures, as demonstrated in the pioneer study of Ni^II^-fluoride complexes that form self-complementary networks held by a NiF···I(C) halogen bond.^21^ To understand how the ^19^F NMR resonances of the nickel-bonded fluoride are affected by the halogen bonds formed in the network, a computational study was performed in the square-planar *trans*-[NiF(2,3,4,5-C_6_F_4_I)(PEt_3_)_2_] (**1oF**), *trans*-[NiF(2,3,5,6-C_6_F_4_I)(PEt_3_)_2_] (**1pF**), and *trans*-[NiF(C_6_F_5_)(PEt_3_)_2_] (**3F**) complexes (Figure 1).^22^ The ^19^F NMR chemical shifts of these complexes were calculated in both the solution and the solid state to investigate the origin of the shielding. Preliminary density functional theory (DFT) calculations, including a continuum solvent model for benzene, reproduced the ^19^F NMR shifts of the nickel-bonded fluorine in **1pF** and **3F** in excellent agreement with the experimental data^23^ (within −0.1 and −2.1 ppm, respectively). However, the chemical shift of **1oF** was not accurately reproduced. In this case, the calculation at the 2c-ZORA-PBE/TZ2P level showed a shift value of ∼23 ppm more shielded than that in the experiment.

As relativistic effects were found to be small,^22^ and such discrepancies clearly exceed the expected margin of error for the functional and basis set,^24^ it was suspected that specific solvent interactions in the experimentally used benzene, which are not adequately covered by the standard implicit solvent model used, are responsible for the differences. Moreover, functional groups may also influence the ^19^F shielding, either through direct noncovalent interactions or through conformation alteration.^11^ Therefore, the influence of both the *ortho* iodine on the nickel-bonded fluoride resonance, which could significantly depend on the motion of the aryl group, and the intramolecular interactions between the fluoride and the phosphine groups is unlikely to be properly represented by the single structure retained for the calculation. In this work, we will demonstrate these factors by employing ab initio molecular dynamics (AIMD) simulations.

## 2 Methods

### 2.1 Structure Optimization

We conducted geometry optimization calculations for **1oF**, **1pF**, and **3F** at the PBE0/TZ2P level of theory.^25−27^ Scalar and spin–orbit relativistic effects at the two-component (2c) level were included using the zeroth-order regular approximation (ZORA) Hamiltonian.^28−32^ To address dispersion effects, we employed Grimme’s D3 approach.^33^ Core electrons were represented using an effective core potential that integrates a small core, as implemented in the *ADF* program, version 2022.^34,35^ Furthermore, we incorporated the COSMO implicit solvent model for benzene during the optimization process.^36−38^

### 2.2 Ab Initio Molecular Dynamics Simulations

Ab initio molecular dynamics (AIMD)^39^ simulations of complex **1oF** were performed in explicit benzene solvation according to the Born–Oppenheimer approximation using the CP2K program package.^40^ The starting model system for AIMD simulations was produced using the Packmol program.^41^ The model consists of complex **1oF** surrounded by 50 benzene molecules in a cubic box of edge 20.4 Å, reproducing the solvent density of 0.87 g/mL. The simulation cell was treated under periodic boundary conditions using a time step of 0.25 fs. The simulation was performed with Kohn–Sham DFT (PBE exchange-correlation functional)^25,26^ and a combined DZVP Gaussian and auxiliary plane-wave (250 Ry cutoff) basis set.^42^ The pure functional PBE was used instead of the hybrid functional PBE0 (used for geometry optimizations) to reduce the computational cost of the AIMD simulations. The core electrons were accounted for using pseudopotentials of the Goedecker–Teter–Hutter (GTH) type.^43^ The dispersion correction was considered with Grimme’s D3 model.^33^

The equilibration of the initial model conformation was performed using a microcanonical ensemble (NVE) until an average temperature of 298 K was reached. After the equilibration, a production trajectory of 30 ps was generated using a canonical (NVT) ensemble with a temperature of 298 K regulated with the CSVR algorithm.^44^ From the simulation of 30 ps, a total of 180 snapshots were taken randomly. Identical snapshots were used for modeling dynamic NMR with and without explicit solvents. The geometries from the AIMD simulation were not optimized further because we are interested in the chemical shifts of the thermodynamic ensemble of structures.

### 2.3 ^19^F NMR Chemical Shift Calculations

We computed the NMR shielding tensors and chemical shifts of the nickel-bound fluorine on different systems, namely, (1) **1oF**, **1pF**, and **3F** complexes, (2) **1oF** with an explicit benzene molecule, (3) snapshots of **1oF** from AIMD simulations without benzene molecules, and (4) snapshots of **1oF** from AIMD simulations with three explicit benzene molecules chosen through noncovalent interaction (NCI) analysis. For the snapshots from AIMD simulations, we computed the final chemical shift value by averaging the values across an ensemble of structures.

The ^19^F NMR calculations were performed with the PBE functional^25,26^ along with the all-electron Slater-type orbitals (STO) TZ2P basis set.^27^ The shielding tensors were computed with an implicit COSMO solvent model for benzene.^36−38^ Relativistic effects were considered using the 2c-ZORA approach,^28−32^ as implemented in the *ADF* program.^34,35^ The gauge-origin problem was treated using the gauge-invariant atomic orbital (GIAO) approach.^45^ Additional static δ(^19^F) calculations were performed for **1oF**, **1pF**, and **3F** complexes using two approaches: (1) at the 2c-ZORA-PBE/TZ2P level in the gas phase to evaluate the performance of the COSMO model and (2) using a nonrelativistic (NR) method at the PBE/TZ2P level to examine the relativity dependence of the 2c-ZORA results. All calculated shieldings σ(^19^F) were converted to chemical shifts δ(^19^F) (in ppm) relative to the shielding of trichlorofluoromethane, computed at the same level of theory (CFCl_3_, calculated σ(^19^F) = 144.0 (2c-ZORA)_solv_, 144.6 (NR)_solv_, 141.6 (2c-ZORA)_gas_).

### 2.4 Noncovalent Interactions Analysis

The noncovalent interactions between **1oF** and explicit solvent molecules were analyzed with the NCIPLOT 4.0 program.^46^ The density and gradient files generated by the program were used to draw the isosurface, displaying the interactions. The VMD program^47^ was used for drawing the isosurface and selecting the solvent molecules interacting closely with **1oF**. The gradient isosurfaces (*s* = 0.3 au) are colored on a blue-green-red scale analogous to the values of sign(λ2)ρ ranging from −3.0 to 3.0 au.

A data set collection of the computational results is available in the *ioChem-BD* repository^48^ and can be accessed via 10.19061/iochem-bd-6-422.

## 3 Results and Discussion

### 3.1 Static Approach for ^19^F NMR Chemical Shifts in Solution

As a first approximation, the ^19^F NMR chemical shifts δ(^19^F) of the nickel-bonded fluoride in complexes **1pF**, **1oF**, and **3F** (Figure 1) were calculated based on *static* (fully optimized) structures using the PBE0 functional, including an implicit COSMO solvation model for benzene; see the Computational Methods section for more details. The structural parameters of the computed structures show minor variations compared to the previously reported values obtained with a PBE0/SMD approach.^22^ For instance, the Ni–F bond distances in **1pF** and **3F** are slightly larger than the ones reported earlier, with differences of 0.014 and 0.015 Å, respectively (Table S1). These differences can be attributed to the modified computational protocol used in this work, which includes changes to the basis set, implicit solvent model, and the consideration of relativistic effects. However, the calculated Ni–F bond distance in **1oF** (1.837 Å) is only 0.003 Å longer than the one obtained at the PBE0/SMD level. Overall, the structural parameters obtained from both methodologies are consistent, allowing us to employ the selected 2c-ZORA-PBE0/TZ2P approach combined with the COSMO model for further calculations of the ^19^F NMR chemical shifts.

For the static δ(^19^F) shift calculations, the performance of the COSMO model vs gas phase was assessed, and the role of relativity was analyzed by comparing the nonrelativistic (NR) approach and the two-component SO relativistic zeroth-order regular approximation (2c-ZORA); see the Computational Methods section for more details. The resulting ^19^F NMR chemical shifts, reported as deviations from the experimental values (Δδ), are shown in Table 1. The sole mismatch in the case of **1oF** is clearly observed in our calculations; at the 2c-ZORA level, the Δδ values are −18.2 (gas phase) and −19.5 ppm (benzene solution). By contrast, **1pF** and **3F** show good agreement with the experimental values. Notably, the computed δ(^19^F) values in the solution phase, particularly those including relativistic effects, outperformed the gas-phase calculations. Moreover, the δ(^19^F) values at the 2c-ZORA level show an improvement of ∼2 and ∼5 ppm over their nonrelativistic values. Although this improvement is relatively modest, the importance of including both scalar and spin–orbit relativistic contributions at the 2c-ZORA level is evident.

**Table 1:** Static ^19^F NMR Chemical Shifts (in ppm) for **1oF**, **1pF**, and **3F** Computed in the Gas Phase and in Benzene Solution, Comparing Both Nonrelativistic (NR) and Two-Component (2c-ZORA) Methods with the Experimental Values

		calculations in gas phase^a^				calculations in benzene solution^a^			
complex	Exp (δ)^b^	NR (δ)	Δδ^c^	2c-ZORA (δ)	Δδ^c^	NR (δ)	Δδ^c^	2c-ZORA (δ)	Δδ^c^
**1oF**	–397.9	–414.6	–16.7	–416.1	–18.2	–413.4	–15.5	–417.4	–19.5
**1pF**	–388.3	–376.4	11.9	–379.2	9.1	–378.6	9.7	–383.7	4.6
**3F**	–394.3	–386.4	7.9	–388.9	5.4	–387.9	6.4	–392.7	1.6

a See Computational Methods section for more details.

b Values reported in ref (21).

c Δδ = δ(calc) – δ(exp).

**Figure 2:**
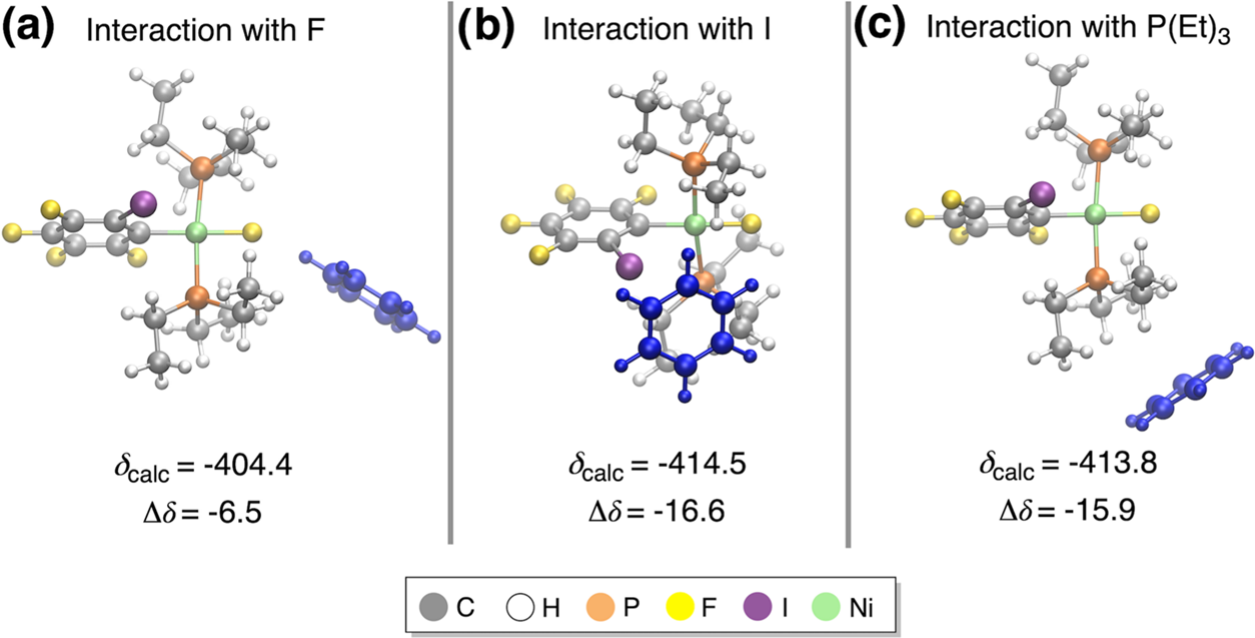
^19^F NMR chemical shifts of complex **1oF** interacting with an explicit benzene molecule in three different positions: (a) near the nickel-bonded fluoride, (b) near the iodine atom of the substituted phenyl ligand, and (c) near one of the triethyl phosphine ligands. The benzene molecule is shown in blue for clarity. The calculated Δδ values (ppm) give the difference between the calculated and experimental chemical shifts.

As a second step in the treatment of the solvation process, we analyzed the effect of adding explicit solute–solvent interactions to the ^19^F chemical shift of **1oF**. To achieve this, we examined the inclusion of a benzene molecule in three different positions around **1oF** (Figure 2): (a) near the nickel-bonded fluoride, (b) near the iodine atom of the substituted phenyl ligand, and (c) near one of the triethyl phosphine ligands. The δ(^19^F) values were calculated at the 2c-ZORA-PBE/TZ2P level. These calculations were based on static (fully optimized) structures using an implicit COSMO solvation model for benzene (see the Computational Methods section).

When comparing the δ(^19^F) values of **1oF** with and without an explicit benzene molecule, the effect of adding a benzene molecule near the iodine atom or the P(Et)_3_ ligand (Figure 2b,c) is rather small (differences up to 3.5 ppm). In contrast, the inclusion of a benzene molecule near the nickel-bonded fluoride (Figure 2a) significantly improved the δ(^19^F) value, reducing the error from −19.5 to −6.5 ppm. This finding corroborates the importance of explicit solute–solvent interactions on the ^19^F NMR chemical shifts, particularly highlighting the need to consider the interaction between fluoride and benzene in the present case.

### 3.2 Influence of Dynamics on the ^19^F NMR Chemical Shifts

To move beyond a static description and provide a more realistic representation of the system’s behavior,^49−56^ we performed AIMD simulations where complex **1oF** was surrounded by solvent molecules (benzene) and followed over time (see Computational Methods section for more details). From these simulations, samples of snapshots were taken and used to obtain dynamically averaged ^19^F NMR chemical shifts. Since these δ(^19^F) values are derived from an ensemble of structures, they can be referred to as *dynamic*^19^F NMR chemical shifts.

**Figure 3:**
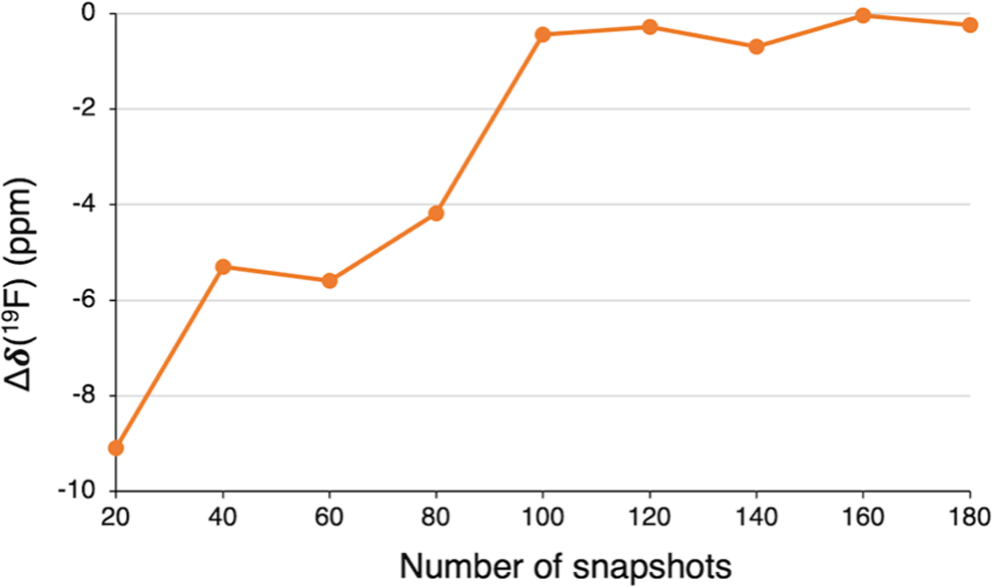
Dynamic ^19^F NMR chemical shift error (Δδ, in ppm) for **1oF**, calculated using different numbers of snapshots along a NVT trajectory of 30 ps. The Δδ values (in ppm) give the difference between calculated and experimental chemical shifts.

We first computed the dynamic δ(^19^F) chemical shifts for the solute alone (complex **1oF**), i.e., removing the solvent molecules from the snapshots before computing the chemical shifts. Thus, these calculations capture only the effect of dynamic motion on the δ(^19^F) values. To ensure convergence in the δ(^19^F) values and avoid bias in the NMR calculations due to an insufficient number of snapshots, we implemented a systematic approach. Thus, from a production NVT trajectory of 30 ps, we first used a sample of 20 random snapshots to calculate the dynamically averaged δ(^19^F) value. We then repeated this procedure, increasing the sample size by 20 snapshots each time until we reached a total of 180 snapshots (Figure 3 and Table S2). These calculations were performed at the 2c-ZORA-PBE/TZ2P level, including the implicit COSMO model for benzene.

As shown in Figure 3, the convergence behavior of the δ(^19^F) values clearly indicates that a large number of snapshots must be taken into account. The dynamic ^19^F NMR chemical shift value reasonably converges to a value of ca. −398 ppm after the first 100 snapshots, with a small error of −0.4 ppm. Beyond this point, only insignificant fluctuations were observed. Notably, the accuracy is drastically improved from a static (−19.5 ppm) to a dynamic approach (−0.4 ppm) using a total of 100 snapshots. Therefore, it is evident that considering the structural flexibility of **1oF** is crucial to obtaining a good agreement with the experimental value.

**Figure 4:**
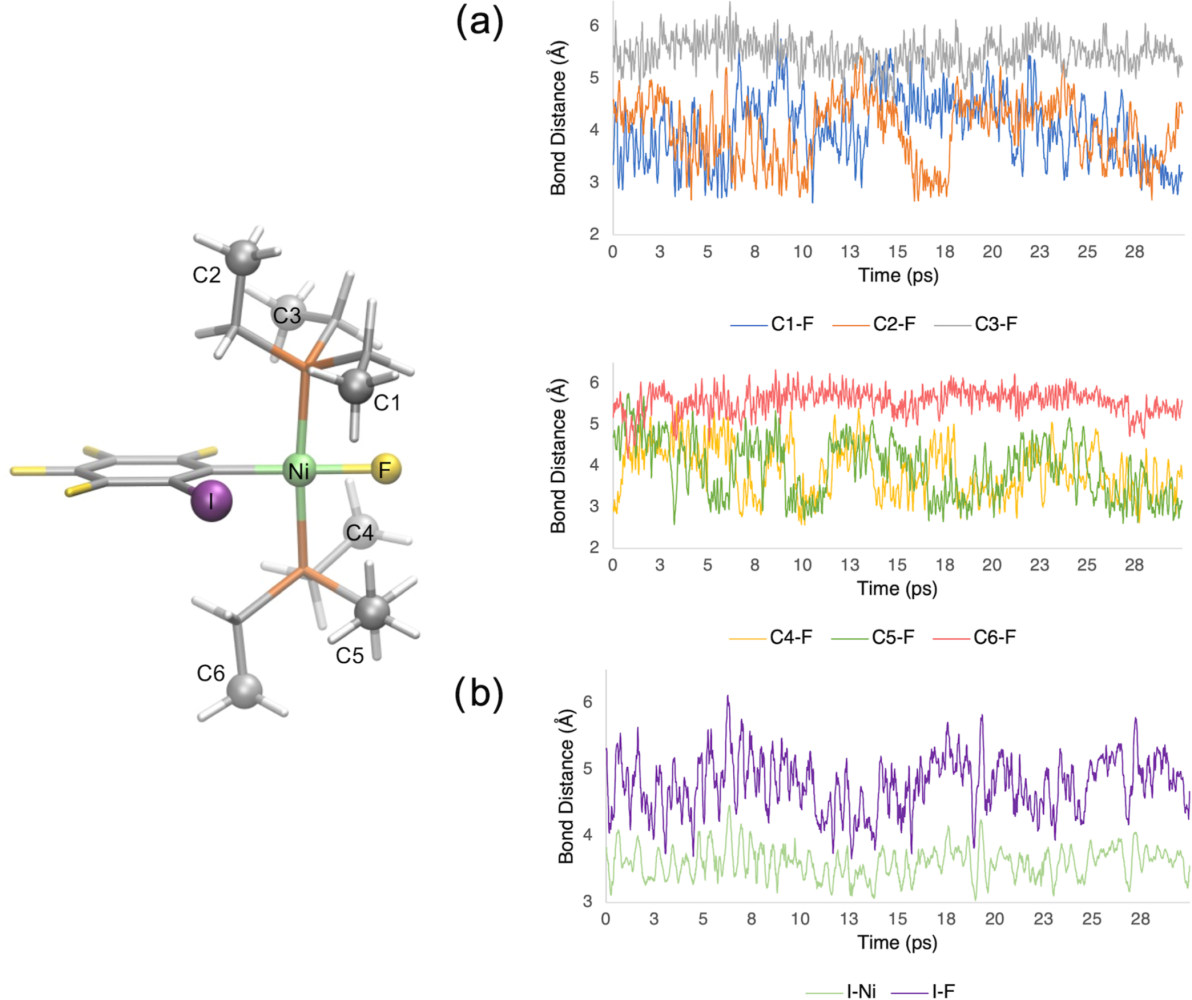
Evolution of distances between (a) the fluoride ligand and the carbon atoms of the PEt_3_ ligands, and (b) the iodine atom on the phenyl ligand with either the nickel metal or the fluoride ligand, along the NVT trajectory of **1oF** complex.

To understand the reasons behind this improved accuracy, we analyzed the molecular dynamics of the **1oF** complex along its trajectory. Notably, the triethyl phosphine (PEt_3_) ligands were demonstrated to be highly fluxional, exhibiting conformational changes that are not considered in the static optimized structures. By analyzing the F···C distances between the nickel-bonded fluoride and the terminal carbon atoms of the ethyl arms (C1–C6) in the PEt_3_ ligand (Figure 4a), we can gain a reasonable description of the intramolecular interactions between the Ni-bonded fluoride and the hydrogen atoms of the PEt_3_ ligands. The large variation in the F···C distances reveals that the carbon atoms, specifically C1, C2, C4, and C5, exhibit significant flexibility and frequently approach the nickel-bonded fluoride, with a minimum F···C distances of around 2.6 Å (Figures 4a, S1a and Table S3).

Furthermore, we observed significant flexibility in the iodine atom on the phenyl ligand during the AIMD trajectory. Notably, this flexibility allows interactions between the iodine atom and both the fluoride ligand and the metal center. To further analyze these interactions, we examined the I···F and I···Ni distances along the trajectory (Figures 4b, S1b, and Table S3). The iodine atom can approach fluorine with a minimum distance of 3.7 Å and an average distance of 4.8 Å. The nickel metal center can make even closer contact with the iodine atom, with a minimum distance of 3.0 Å and an average distance of 3.6 Å. This indicates the presence of short I···Ni interactions, where the distance between atoms is less than the sum of Bondi’s van der Waals radii (3.61 Å).^57^ Such interactions may be identified as boundary noncovalent interactions.^58^ Thus, these findings highlight the significant role of noncovalent interactions, attributed to the considerable flexibility of both the phenyl ring and the two phosphine ligands in the complex, in determining the ^19^F NMR chemical shifts of **1oF**. It is worth noting that a similar flexibility for phosphine ligands is also observed in the **1pF** complex (Figure S2 and Table S4). However, in **1pF**, the iodine atom is in the *para* position to Ni, restricting interactions between I···F and I···Ni, which reduces the phenyl ring’s flexibility compared to **1oF**. This suggests fewer dynamic effects on **1pF** and aligns with the results of the static NMR calculations, as they successfully reproduced the chemical shifts of **1pF** and **3F** but not **1oF**. We will consider a dynamic NMR approach for **1pF** and **3F** in future studies to gain deeper insights into how these interactions affect the shift values.

**Figure 5:**
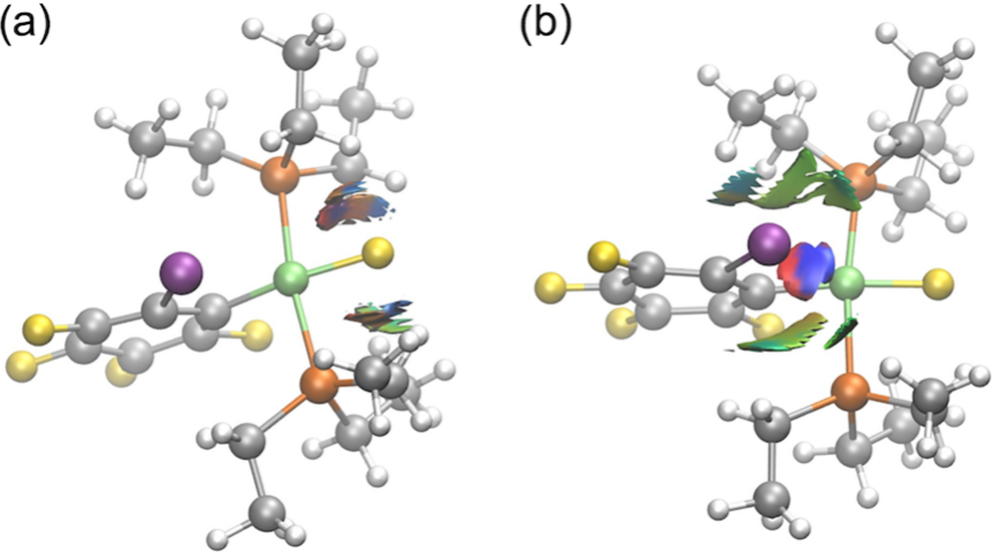
Noncovalent interactions of **1oF** identified close to (a) the nickel-bonded fluoride and (b) the iodine atom on the phenyl ligand. Regions in blue/green indicate strong, attractive interactions, and regions in red indicate strong nonbonded overlap.

To further enhance our understanding of the intramolecular interactions that are present in **1oF**, we carried out a detailed analysis of the noncovalent interactions using the NCIPLOT program (see more details in the Computational Methods section). Specifically, we selected random snapshots where close interactions were observed in the nickel-bonded fluoride or in the iodine atom (Figure 5). This allowed us to identify attractive and repulsive interactions between fluoride and the phosphine ligands (Figure 5a, represented by blue and red colors), agreeing with the earlier observation that the PEt_3_ ligands closely approach fluoride. Comparatively, weak van der Waals interactions are observed between PEt_3_ and fluoride ligands in the optimized geometry of **1oF**, which was used for static NMR calculations (Figure S3a).

The NCI analysis also reveals considerable strong interactions of iodine with the phosphine ligands and the nickel metal center (Figure 5b). The phosphine ligands mainly form weak van der Waals interactions (in green), whereas the nickel engages in strong, attractive interactions (in blue) and some strong repulsive interactions (in red). In contrast, the static optimized geometry of **1oF** shows relatively weaker interactions between the nickel and iodine atoms (Figure S4a). These findings highlight the necessity of a dynamic treatment of the **1oF** complex to capture these significant noncovalent interactions.

### 3.3 Specific Solute–Solvent Interactions on the Dynamic ^19^F NMR Chemical Shifts

**Figure 6:**
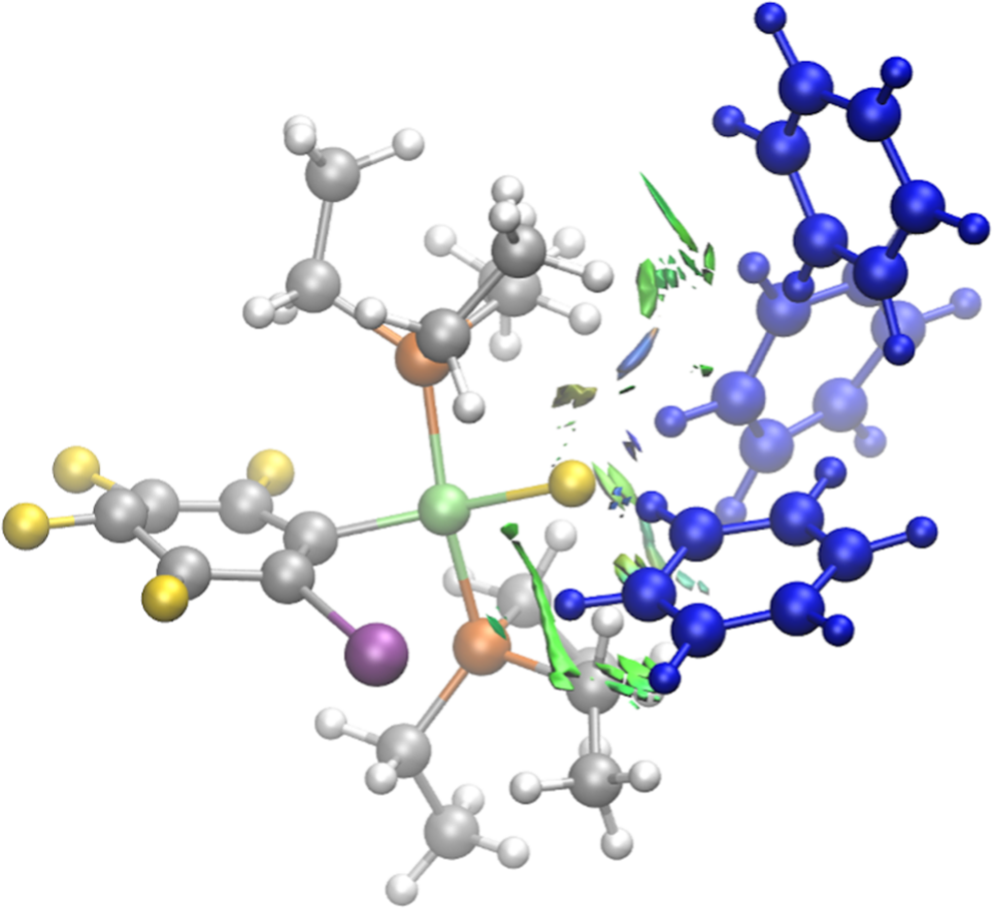
AIMD snapshot of complex **1oF** with three explicit benzene molecules identified based on the noncovalent interaction regions using the NCIPLOT program. Regions in blue/green indicate strong, attractive interactions, and regions in red indicate strong nonbonded overlap. The selected three benzene molecules are shown in blue for clarity.

As an additional step in the solvation process, we examined the impact of incorporating explicit benzene molecules on dynamic ^19^F NMR chemical shift calculations. To achieve this, we selected three benzene molecules per snapshot. This approach allowed us to focus on solvent molecules that directly interact with **1oF** while maintaining computational efficiency. The identification of which solvent molecules to include is, however, not straightforward and we resorted to the NCIPLOT program. We gave priority to the solvent molecules surrounding the fluoride atom based on our static NMR results, where the inclusion of one explicit solvent molecule yielded the best accuracy when interacting with the fluoride ligand (Figure 2). For each snapshot, we manually selected the three benzene molecules that exhibited the most substantial noncovalent interactions (see Figure 6; strong interactions are indicated in blue, and weaker dispersion interactions in green).

**Figure 7:**
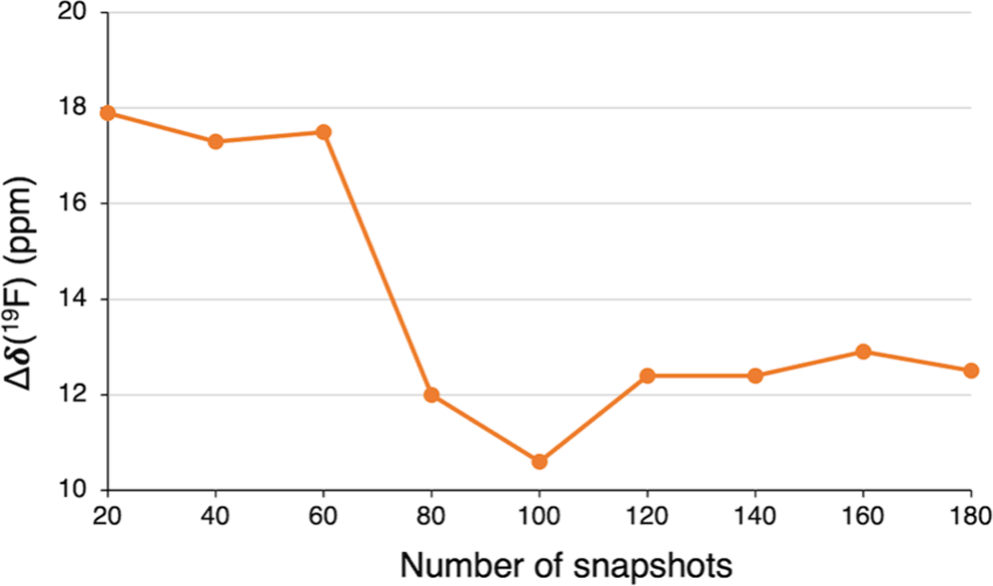
Dynamic ^19^F NMR chemical shift error (Δδ, in ppm) for **1oF** interacting with three benzene solvent molecules, calculated using a different number of snapshots along a NVT trajectory of 30 ps. The Δδ values (in ppm) give the difference between calculated and experimental chemical shifts.

The dynamically averaged ^19^F NMR chemical shifts of **1oF** with three explicit benzene molecules were calculated using the systematic approach described in Section 3.2. Thus, we collected samples of 20 random snapshots, increasing the sample size by 20 snapshots each time until we reached a total of 180 snapshots (Figure 7 and Table S5). The dynamic ^19^F NMR chemical shift value reasonably converges to a value of ca. −385.5 ppm after the first 120 snapshots, having an error of 12.4 ppm, with minor fluctuations observed beyond this point. The inclusion of explicit benzene molecules in the dynamic approach causes a large deshielding on the nickel-bonded fluoride, resulting in an increase of approximately 13 ppm in the δ(^19^F) value. As a result, this leads to a larger deviation from the experimental value by 12.4 ppm, which is less accurate compared to the dynamic NMR calculations performed without explicit solvent molecules (Figure 3 and Table S2). Hence, these findings suggest that the inclusion of explicit benzene molecules appears to deteriorate the dynamic δ(^19^F) results. It is known that variables such as the number of explicit solvent molecules and their strategic placement can critically influence the precision of computational outcomes.^59−62^ Thus, it appears that our approach has been adversely affected by the limited selection of only three benzene molecules, which may not adequately account for the solute–solvent interactions affecting the computed chemical shift values. Moreover, the selection of explicit solvent molecules only around the fluoride ligand appears to overestimate the deshielding effect. These interpretations need to be assessed by considering a larger selection of solvent molecules to complete the first full solvation shell around the complex. However, this approach was not implemented due to the high associated computational cost.

### 3.4 Static vs Dynamic Treatment

After the detailed examination of the solvation effects, a general comparison of the results can be made to determine the most effective approach for the prediction of δ(^19^F) in the **1oF** complex (Table 2). Our first static approach, considering an isolated **1oF** complex in the gas phase, showed a change of −18.2 ppm when compared to the experimental value. The inclusion of the COSMO model resulted in minor changes, indicating that the electronic solute–solvent interactions cannot be properly modeled with a continuum model. However, such a model is useful for describing bulk solvent effects. Notably, the addition of an explicit solvent benzene molecule near the fluoride ligand has a direct effect on the δ(^19^F) value, resulting in a deviation of −6.5 ppm. However, it is important to mention that this improvement in accuracy was not observed when considering interactions between benzene and iodine or P(Et)_3_ ligands.

**Table 2:** Summary of Computed ^19^F NMR Chemical Shifts (in ppm) for the Nickel-Bonded Fluoride in **1oF**, Using Both Static and Dynamic Approaches and Comparing with the Experimental Value

		chemical shift (δ)	Δδ^a^
experimental shift value^b^		–397.9	
static-gas phase	isolated **1oF** complex	–416.1	–18.2
static-COSMO	isolated **1oF** complex	–417.4	–19.5
static-COSMO	**1oF** + 1 benzene^c^	–414.5	–16.6
static-COSMO	**1oF** + 1 benzene^d^	–413.8	–15.9
static-COSMO	**1oF** + 1 benzene^e^	–404.4	–6.5
dynamic	isolated **1oF** complex	–398.1	–0.2
dynamic	**1oF** + 3 benzene	–385.4	12.5

a Δδ = δ(calc) – δ(exp).

b Value reported in ref (21).

c Interaction of benzene with I.

d Interaction of benzene with P(Et)_3_ ligand.

e Interaction of benzene with fluoride ligand.

In the dynamic approach, it is crucial to emphasize the importance of including dynamic averaging in the calculation of the ^19^F NMR chemical shift. This involves calculating an averaged δ(^19^F) value by taking snapshots from the AIMD trajectory and considering the isolated **1oF** complex as the basis for δ(^19^F) calculations. Both the vibrational averaging over the molecule’s degrees of freedom at room temperature and the indirect effect induced by the presence of the solvent molecules in the AIMD simulations contribute to understand this phenomenon. The effect of dynamical averaging directly affects the chemical shift, which increases by approximately 19 ppm when compared with the static-COSMO approach (Table 2). Furthermore, the addition of explicit solvent molecules enhances this effect. The δ(^19^F) value is further increased but shows a higher error of about 12.5 ppm. As mentioned previously, this is likely due to the inappropriate selection of the benzene molecules from the snapshots. Hence, the best match to the experimental value, with a difference of only −0.2 ppm, is obtained by employing a dynamic approach without including explicit solvent molecules.

## 4 Conclusions

In this study, we examined the reliability of various levels of theory for modeling the ^19^F NMR chemical shifts in solution for square-planar nickel-fluoride complexes. The calculation of these chemical shifts presents a challenge in computational chemistry, mainly due to solvent effects, which complicates the establishment of a simple protocol for conducting such studies. Particularly, we focused on the *trans*-[NiF(2,3,4,5-C_6_F_4_I)(PEt_3_)_2_] (**1oF**), *trans*-[NiF(2,3,5,6-C_6_F_4_I)(PEt_3_)_2_] (**1pF**), and *trans*-[NiF(C_6_F_5_)(PEt_3_)_2_] (**3F**) complexes. The modeling of the δ(^19^F) values for **1pF** and **3F** species was successful by using a static approach with an implicit solvation model. However, this approach failed for the **1oF** complex, which showed a large discrepancy (∼20 ppm) compared with the experimental signal. To address this discrepancy, we first investigated a static protocol, including specific solute–solvent interactions on the ^19^F NMR calculations. Notably, the interaction of a benzene molecule with the nickel-bonded fluoride caused a large deshielding effect, significantly improving the description of the δ(^19^F) value.

More advanced dynamic protocols were also employed to calculate the δ(^19^F) values. These protocols involved AIMD simulations of the complex **1oF** surrounded by explicit benzene solvent molecules. In these calculations, we initially selected random snapshots and considered the isolated **1oF** complex to calculate an averaged (^19^F) value. Subsequently, we introduced the inclusion of three benzene molecules to examine their impact. A careful monitoring of the number of snapshots was crucial to ensure convergence of the (^19^F) values. The convergence of the dynamic ^19^F NMR chemical shifts was achieved at 100 snapshots for the approach using the isolated **1oF** complex and at 120 snapshots for the approach including **1oF** and three explicit benzene molecules.

The inclusion of dynamic averaging using AIMD simulations resulted in a significant improvement in the ^19^F NMR chemical shift. This improvement can be attributed to the large flexibility exhibited by both the phenyl and the two P(Et)_3_ ligands within the complex, which results in significantly noncovalent interactions around the fluoride ligand and nickel metal center during the AIMD trajectory. Including these interactions with molecular dynamics resulted in the precise calculation of the chemical shift for **1oF**. Notably, these interactions are missing in **1pF** and **3F**, as the iodine is too far from nickel in the former and absent in the latter. In contrast, the inclusion of three explicit benzene molecules on the dynamic NMR calculations caused a large deshielding of 12.5 ppm. This deshielding was likely caused by the interactions between benzene and fluoride, which were the focus when selecting the three explicit benzene molecules, potentially skewing the averaged chemical shift value. Overall, our study demonstrates that accounting for dynamic conformational flexibility using AIMD simulations can result in accurate ^19^F NMR chemical shift calculations, rationalizing the use of advanced quantum chemical methods for calculating NMR resonances. Hence, this study reports an important protocol for the ^19^F NMR characterization of nickel-fluoride complexes in solution.

## Supporting Information Available

Additional computational data of static and dynamic ^19^F NMR chemical shifts; histogram plots of distances; additional figures of noncovalent interactions (PDF)
Cartesian coordinates (XYZ)

## Supplementary Material

jp4c05408_si_001.pdf

jp4c05408_si_002.xyz
