# Supplementary material for: Advancing 19F NMR Prediction of Metal-Fluoride Complexes in Solution: Insights from Ab Initio Molecular Dynamics
Source: J Phys Chem A. 2024 Dec 3;128(49):10498–506. doi: 10.1021/acs.jpca.4c05408 (PMC11647890; doi:10.1021/acs.jpca.4c05408)
Supplement: Supplementary file 1 — jp4c05408_si_001.pdf [file jp4c05408_si_001.pdf]

Supporting Information for

Advancing ^{19}F NMR Prediction of Metal-Fluoride Complexes in Solution: Insights from Ab Initio Molecular Dynamics

Sahil Gahlawat,[a,b] *Kathrin H. Hopmann,*[a] *and Abril C. Castro** [c]

[a]Department of Chemistry, UiT The Arctic University of Norway, 9037 Tromsø, Norway

[b]Hylleraas Centre for Quantum Molecular Sciences, Department of Chemistry, UiT The Arctic University of Norway, 9037 Tromsø, Norway

[c]Hylleraas Centre for Quantum Molecular Sciences, Department of Chemistry, University of Oslo, 0315 Oslo, Norway

Corresponding Author:

*Abril C. Castro, e-mail: abril.castro@kjemi.uio.no

Table of Contents

Table S1. Selected bond distances (Å) for **1oF**, **1pF**, and **3F** optimized using the COSMO implicit solvent model for benzene. S3

Table S2. The dynamically averaged $\delta(^{19}F)$ values for **1oF** without considering explicit benzene molecules S3

Table S3. Average, minimum, and maximum distances (Å) between fluoride ligand and selected atoms of the complex, as well as between the iodine atom and the nickel metal along the AIMD trajectory of **1oF**. S4

Figure S1. Histogram plots of distances between a) the fluoride ligand and the carbon atoms of the PEt_3 ligands, and b) the iodine atom on the phenyl ligand with either the nickel metal or the fluoride ligand, along the NVT trajectory of **1oF** complex. S5

Figure S2. Evolution of distances between the fluoride ligand and the carbon atoms of the PEt_3 ligands along the NVT trajectory of **1pF** complex S6

Table S4. Average, minimum, and maximum distances (Å) between the fluoride ligand and the carbon atoms of the PEt_3 ligands along the AIMD trajectory of **1pF**. S6

Figure S3. Non-covalent interactions made by a) nickel-bonded fluoride and b) the iodine atom on the phenyl ligand for the optimized geometry of **1oF**.. .... S7

Figure S4. Non-covalent interactions made by nickel-bonded fluoride for the optimized geometries of a) **1pF** and b) **3F** complexes.. .... S7

Table S5. The dynamically averaged $\delta(^{19}F)$ values for **1oF** with explicit three benzene molecules. S8

Figure S5. Workflow for the modeling of static and dynamic ^{19}F NMR chemical shifts......... S9

Table S6. The random snapshots of **1oF** considered for calculating the dynamic ^{19}F NMR chemical shifts (in ppm) with and without the presence of explicit solvent molecules. S10

Figure S6. The plot of dynamic chemical shift values against the snapshot number.. .... S14

Table S1. Selected bond distances (Å) for **1oF**, **1pF**, and **3F** optimized using the COSMO implicit solvent model for benzene.

	Optimized structures in benzene solution[a]		
	**1oF**	**1pF**	**3F**
**Ni–F**	1.8369	1.8419	1.8512
**C–I**	2.1137	2.0927	---
**Ni–P1**	2.1995	2.2042	2.1999
**Ni–P2**	2.2064	2.2043	2.1999
**Ni–C**	1.8870	1.8914	1.8961
**F–Ni–C**	178.295	179.976	179.878
**P1–Ni–P2**	173.932	179.098	172.256
**P1–Ni–F**	82.526	89.549	86.138

[a] The geometries are optimized at PBE0/TZ2P level of theory using the ZORA Hamiltonian.

Table S2. The dynamically averaged δ(^{19}F) values for **1oF** without considering explicit benzene molecules; snapshots are systematically incremented by 20 and until a total of 180 random snapshots.

**Number of snapshots**	$\boldsymbol{\delta}$**(^{19}F)**	$\boldsymbol{\Delta\delta}$[a]
20	-407.0	-9.1
40	-403.2	-5.3
60	-403.5	-5.6
80	-402.1	-4.2
100	-398.3	-0.4
120	-398.2	-0.3
140	-398.6	-0.7
160	-397.9	0.0
180	-398.1	-0.2

[a]$\Delta\delta = \delta$(calc) – δ(exp).

Table S3. Average, minimum, and maximum distances (Å) between fluoride ligand and selected atoms of the complex, as well as between the iodine atom and the nickel metal along the AIMD trajectory of **1oF**. The distances corresponding to the static optimized geometry of **1oF** are also shown.

Interacting atoms	Average distance	Minimum distance	Maximum distance	Optimized distance[a]
C1-F	4.1	2.6	5.9	3.4
C2-F	4.0	2.6	5.4	3.4
C3-F	5.5	4.6	6.5	5.6
C4-F	3.9	2.6	5.5	3.6
C5-F	3.9	2.6	5.8	4.4
C6-F	5.6	4.2	6.3	5.6
I-F	4.8	3.7	6.1	4.7
I-Ni	3.6	3.0	4.5	3.5

[a]Distances corresponding to the static optimized geometry of **1oF**.

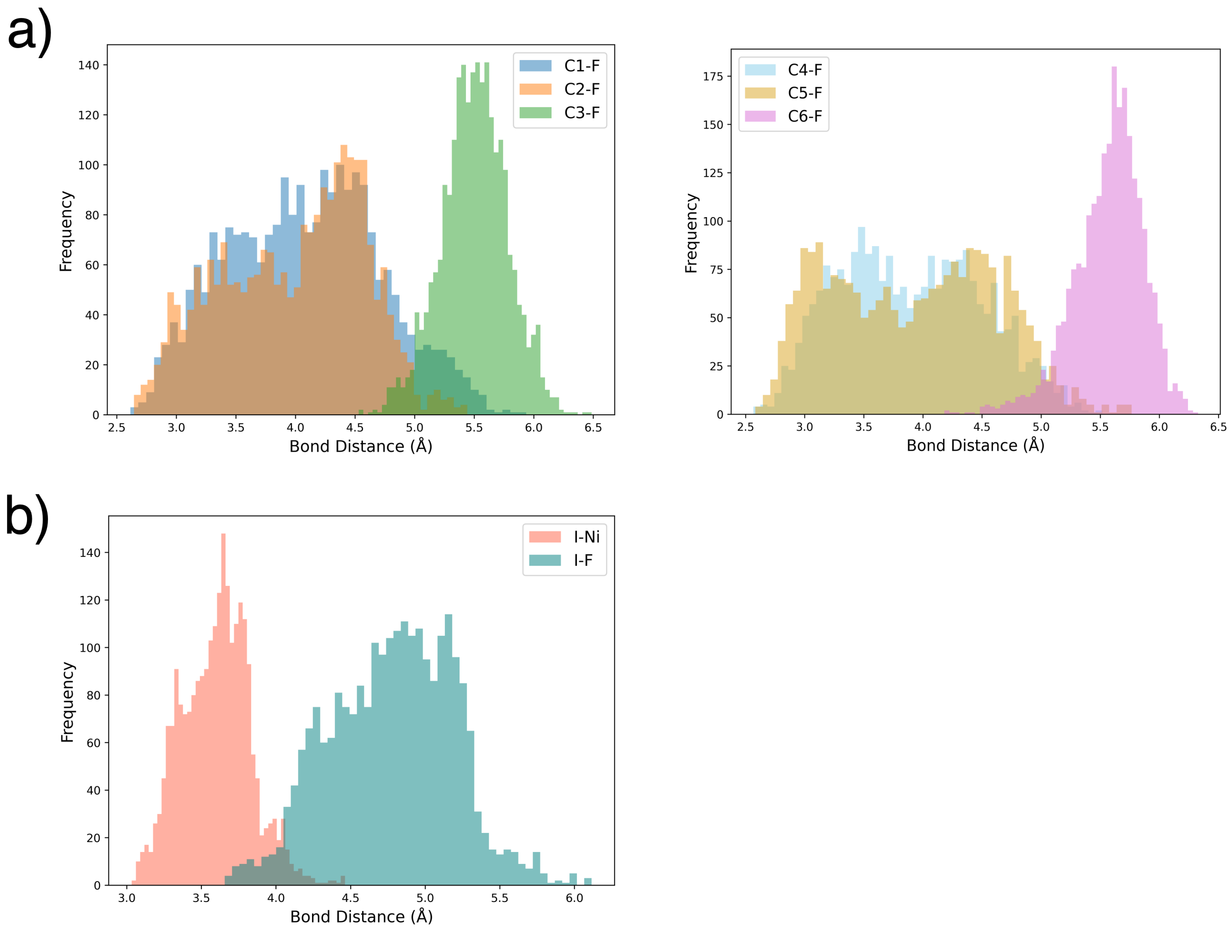

Figure S1. Histogram plots of distances between a) the fluoride ligand and the carbon atoms of the PEt_3 ligands, and b) the iodine atom on the phenyl ligand with either the nickel metal or the fluoride ligand, along the NVT trajectory of **1oF** complex.

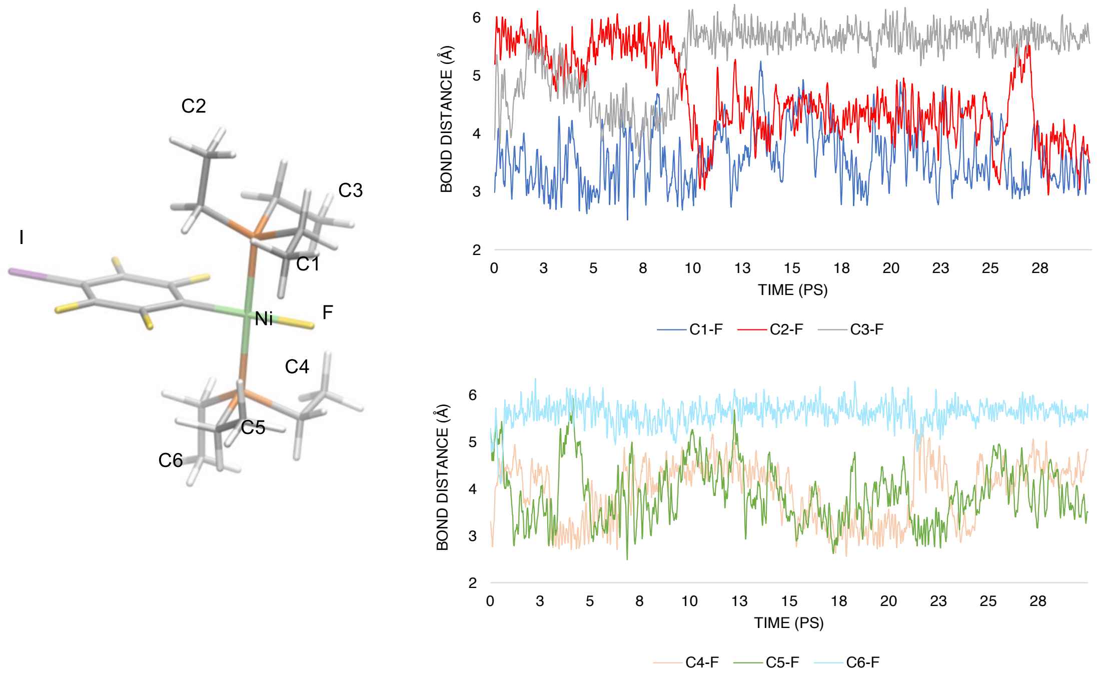

Figure S2. Evolution of distances between the fluoride ligand and the carbon atoms of the PEt_3 ligands along the NVT trajectory of **1pF** complex.

Table S4. Average, minimum, and maximum distances (Å) between the fluoride ligand and the carbon atoms of the PEt_3 ligands along the AIMD trajectory of **1pF**. The distances corresponding to the static optimized geometry of **1pF** are also shown.

Interacting atoms	Average distance	Minimum distance	Maximum distance	Optimized distance[a]
C1-F	3.6	2.5	5.3	3.8
C2-F	4.6	2.9	6.1	3.6
C3-F	5.3	3.6	6.2	4.8
C4-F	3.9	2.6	5.4	3.4
C5-F	3.9	2.5	6.0	3.5
C6-F	5.6	4.1	6.4	5.6

[a]Distances corresponding to the static optimized geometry of **1pF**.

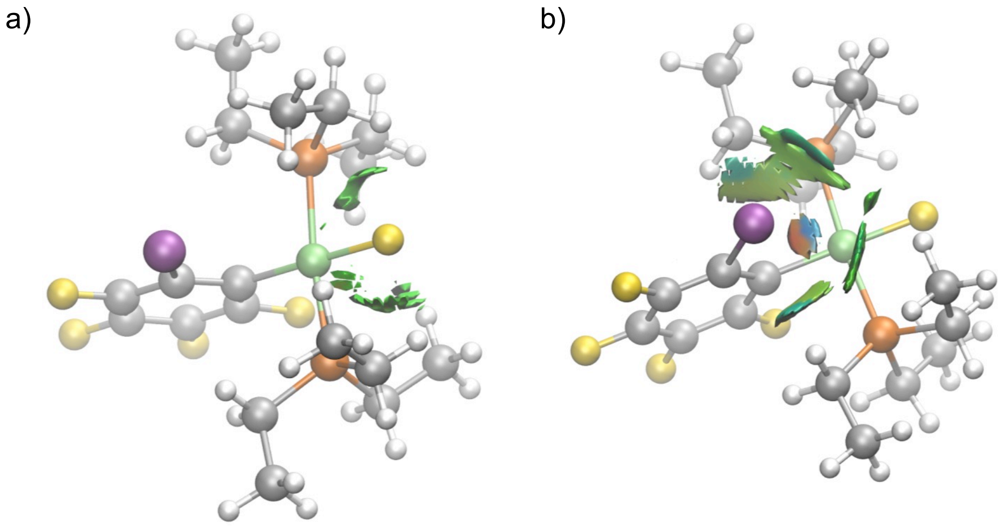

Figure S3. Non-covalent interactions made by a) nickel-bonded fluoride and b) the iodine atom on the phenyl ligand for the optimized geometry of **1oF**. Blue indicates strong, attractive interactions, and red indicates strong nonbonded overlap.

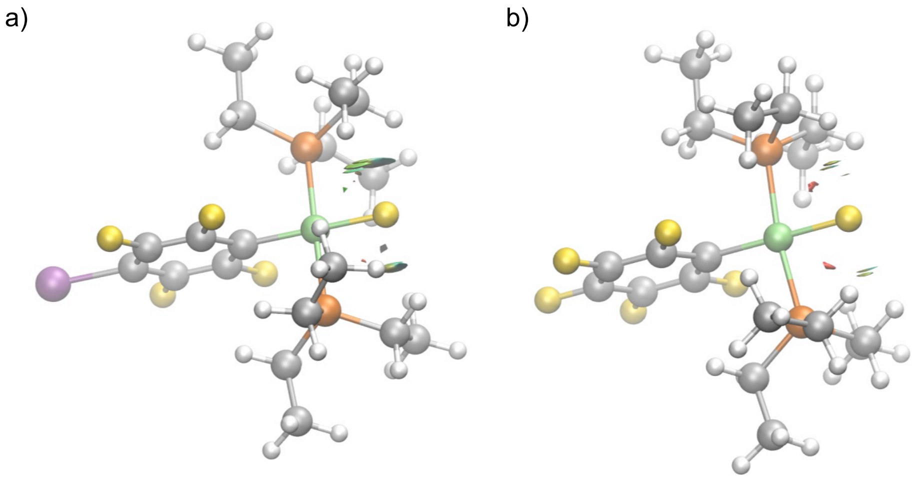

Figure S4. Non-covalent interactions made by nickel-bonded fluoride for the optimized geometries of a) **1pF** and b) **3F** complexes. Blue indicates strong, attractive interactions, and red indicates strong nonbonded overlap.

Table S5. The dynamically averaged $\delta(^{19}F)$ values for **1oF** with explicit three benzene molecules.

Snapshots are systematically incremented by 20 and until a total of 180 random snapshots.

**Number of snapshots**	$\boldsymbol{\delta(^{19}F)}$	$\boldsymbol{\Delta\delta}$[a]
20	-380.0	17.9
40	-380.6	17.3
60	-380.4	17.5
80	-385.9	12.0
100	-387.3	10.6
120	-385.5	12.4
140	-385.5	12.4
160	-385.0	12.9
180	-385.4	12.5

[a] $\Delta\delta = \delta(\text{calc}) - \delta(\text{exp})$.

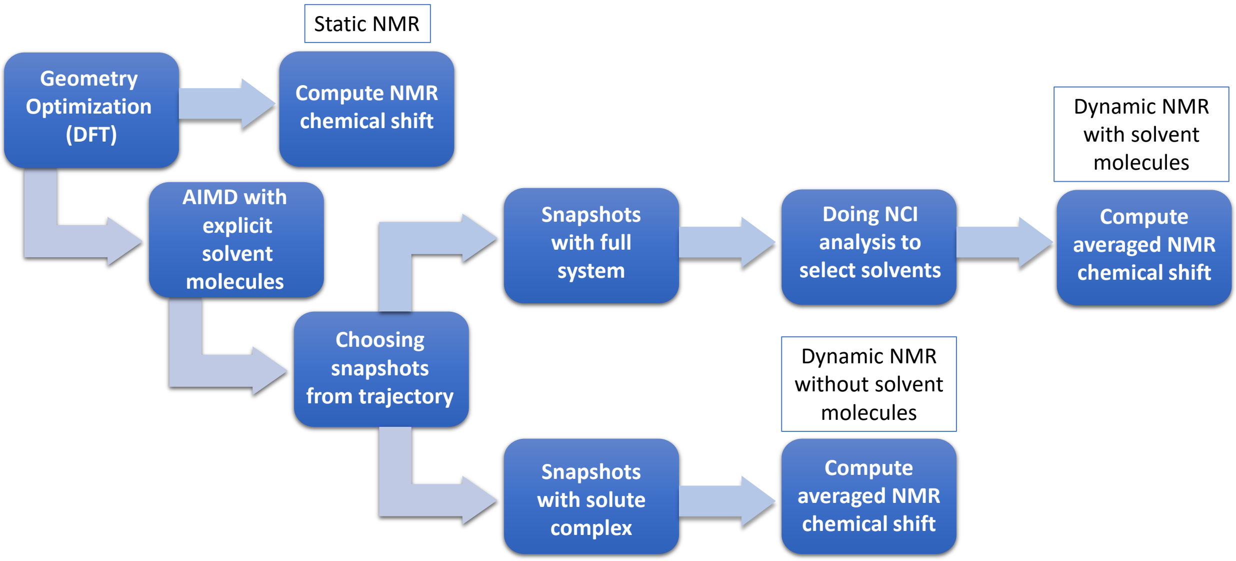

Figure S5. Workflow for the modeling of static and dynamic ^{19}F NMR chemical shifts.

We computed the ^{19}F NMR chemical shift values of nickel-bound fluoride atom in **1oF** in three ways, which are, 1) static NMR, 2) dynamic NMR without solvent molecules, and 3) dynamic NMR with solvent molecules. The workflow of three methods of computing chemical shifts is sown in Figure S1. The static NMR calculation included optimizing **1oF** and the then computing its chemical shift value based on a single structure. On the other hand, average of chemical shift values calculated from an ensemble of structures obtained through AIMD was used for dynamic NMR. We considered the snapshots both with and without the explicit solvent molecules when calculating the averaged chemical shift. Therefore, we have two variations of the dynamic method, NMR with and without explicit solvent molecules. For dynamic NMR with explicit solvents, we choose three relevant benzene molecules interacting closely with **1oF** analyzed using the non-covalent interactions.

Table S6. The random snapshots of **1oF** considered for calculating the dynamic ^{19}F NMR chemical shifts (in ppm) with and without the presence of explicit solvent molecules.

	Shielding constants (σ)		Chemical Shifts (δ)		$\Delta\delta$
**Snapshot number**	**Isolated 1oF**	**1oF + 3 benzene**	**Isolated 1oF**	**1oF + 3 benzene**	
1	632.5	627.3	-488.5	-483.3	5.2
2	475.2	461.1	-331.2	-317.0	14.1
3	596.8	593.9	-452.8	-449.9	2.9
4	548.1	541.2	-404.1	-397.2	6.9
5	521.0	469.7	-377.0	-325.7	51.3
6	528.8	516.4	-384.8	-372.4	12.4
7	545.4	523.7	-401.4	-379.7	21.7
8	563.7	535.4	-419.7	-391.4	28.2
9	582.6	567.5	-438.6	-423.5	15.1
10	577.7	560.6	-433.7	-416.6	17.1
11	342.6	312.7	-198.6	-168.7	29.8
12	564.2	557.6	-420.1	-413.6	6.5
13	467.4	450.6	-323.4	-306.6	16.8
14	555.4	549.3	-411.4	-405.3	6.1
15	548.4	535.5	-404.4	-391.5	12.9
16	503.7	489.2	-359.7	-345.2	14.5
17	506.8	495.1	-362.8	-351.1	11.7
18	510.8	490.7	-366.8	-346.7	20.1
19	419.0	386.5	-275.0	-242.5	32.6
20	764.1	728.8	-620.1	-584.7	35.4
21	502.2	498.2	-358.1	-354.2	3.9
22	497.8	491.8	-353.8	-347.8	6.0
23	556.3	526.1	-412.3	-382.1	30.2
24	610.5	584.6	-466.5	-440.6	25.9
25	608.8	604.4	-464.8	-460.3	4.5
26	587.6	582.8	-443.6	-438.7	4.8
27	621.3	620.6	-477.3	-476.6	0.7
28	590.0	587.2	-446.0	-443.2	2.8
29	622.8	620.3	-478.8	-476.3	2.6
30	564.2	559.7	-420.2	-415.7	4.5
31	536.3	526.2	-392.3	-382.2	10.1
32	690.3	684.8	-546.3	-540.8	5.5
33	428.3	416.0	-284.3	-272.0	12.3
34	555.9	536.3	-411.9	-392.3	19.6
35	627.1	620.4	-483.1	-476.4	6.7
36	544.1	540.8	-400.1	-396.8	3.3
37	569.2	561.7	-425.2	-417.7	7.5
38	553.4	542.3	-409.4	-398.3	11.1

39	539.7	526.2	-395.7	-382.2	13.5
40	563.0	556.3	-419.0	-412.3	6.7
41	489.0	488.4	-345.0	-344.4	0.7
42	551.8	550.5	-407.8	-406.5	1.3
43	510.6	498.4	-366.5	-354.4	12.2
44	525.4	507.6	-381.4	-363.6	17.8
45	615.7	589.2	-471.7	-445.1	26.6
46	583.9	571.5	-439.9	-427.4	12.4
47	522.5	510.7	-378.5	-366.7	11.8
48	493.9	491.0	-349.9	-347.0	2.9
49	502.2	497.9	-358.2	-353.9	4.3
50	462.4	459.4	-318.4	-315.4	3.0
51	558.0	552.4	-414.0	-408.4	5.7
52	489.7	469.6	-345.7	-325.5	20.2
53	488.5	472.4	-344.5	-328.3	16.2
54	616.4	606.0	-472.4	-462.0	10.5
55	522.6	502.8	-378.6	-358.8	19.8
56	514.3	500.7	-370.3	-356.7	13.5
57	523.1	502.8	-379.1	-358.8	20.3
58	529.1	497.1	-385.1	-353.1	32.0
59	534.1	529.3	-390.1	-385.2	4.9
60	634.9	630.2	-490.9	-486.2	4.7
61	586.4	575.9	-442.3	-431.9	10.5
62	528.3	513.7	-384.3	-369.7	14.6
63	500.3	495.1	-356.2	-351.1	5.2
64	477.4	461.2	-333.4	-317.2	16.2
65	569.9	560.0	-425.9	-416.0	9.9
66	616.6	600.9	-472.6	-456.9	15.7
67	480.5	467.0	-336.5	-323.0	13.5
68	545.1	540.0	-401.1	-396.0	5.2
69	546.7	532.8	-402.7	-388.8	13.9
70	566.0	562.4	-422.0	-418.4	3.6
71	589.1	578.6	-445.0	-434.6	10.5
72	514.3	493.1	-370.3	-349.1	21.2
73	548.7	529.3	-404.7	-385.2	19.5
74	491.5	464.1	-347.5	-320.1	27.4
75	655.4	650.9	-511.4	-506.9	4.6
76	463.1	457.4	-319.1	-313.3	5.7
77	557.0	549.8	-412.9	-405.8	7.2
78	618.8	572.0	-474.7	-428.0	46.7
79	547.4	532.0	-403.4	-388.0	15.4
80	536.4	520.4	-392.4	-376.4	16.0
81	558.0	552.1	-414.0	-408.1	5.9
82	604.1	601.1	-460.1	-457.1	3.0
83	487.7	487.4	-343.7	-343.3	0.3

84	639.0	628.5	-495.0	-484.5	10.5
85	570.6	548.7	-426.5	-404.7	21.8
86	529.9	522.2	-385.9	-378.2	7.7
87	471.0	450.8	-327.0	-306.8	20.2
88	500.1	468.3	-356.1	-324.3	31.8
89	532.6	519.2	-388.6	-375.1	13.5
90	497.5	465.5	-353.5	-321.5	32.0
91	569.8	557.9	-425.8	-413.9	11.9
92	441.0	407.7	-297.0	-263.7	33.3
93	581.7	572.6	-437.7	-428.6	9.0
94	541.2	527.8	-397.2	-383.8	13.4
95	484.5	466.9	-340.5	-322.9	17.6
96	597.9	573.3	-453.9	-429.3	24.6
97	551.2	543.3	-407.2	-399.3	7.8
98	525.6	516.4	-381.6	-372.3	9.2
99	533.7	525.4	-389.7	-381.3	8.4
100	560.5	537.5	-416.5	-393.4	23.0
101	487.3	483.2	-343.3	-339.2	4.1
102	516.8	511.7	-372.7	-367.7	5.0
103	546.7	538.2	-402.7	-394.2	8.5
104	560.2	552.1	-416.2	-408.1	8.1
105	576.9	569.7	-432.9	-425.6	7.2
106	535.8	516.3	-391.8	-372.3	19.5
107	596.3	589.6	-452.2	-445.6	6.6
108	443.2	424.2	-299.2	-280.2	19.0
109	559.2	548.7	-415.2	-404.6	10.5
110	487.4	481.0	-343.4	-337.0	6.4
111	581.3	569.8	-437.3	-425.8	11.5
112	560.3	557.6	-416.3	-413.6	2.7
113	559.1	547.5	-415.1	-403.4	11.6
114	550.7	535.8	-406.7	-391.8	14.9
115	540.0	536.2	-396.0	-392.2	3.9
116	579.4	576.6	-435.4	-432.6	2.8
117	532.4	527.8	-388.4	-383.8	4.5
118	464.1	438.0	-320.0	-294.0	26.0
119	658.6	632.1	-514.5	-488.1	26.5
120	494.3	484.4	-350.3	-340.4	9.9
121	568.5	557.7	-424.5	-413.7	10.7
122	516.0	502.3	-372.0	-358.3	13.6
123	491.7	472.8	-347.7	-328.8	18.9
124	582.6	563.7	-438.6	-419.7	18.9
125	586.5	574.0	-442.5	-430.0	12.5
126	467.9	451.6	-323.9	-307.6	16.3
127	478.5	455.9	-334.5	-311.9	22.6
128	602.9	592.1	-458.8	-448.1	10.7

129	457.9	439.9	-313.9	-295.9	18.1
130	544.2	530.1	-400.1	-386.1	14.1
131	461.5	433.3	-317.4	-289.2	28.2
132	560.4	544.9	-416.4	-400.9	15.5
133	519.0	498.2	-375.0	-354.2	20.7
134	518.5	504.0	-374.5	-360.0	14.5
135	594.3	586.3	-450.3	-442.3	7.9
136	517.9	502.6	-373.9	-358.6	15.2
137	474.0	468.3	-330.0	-324.3	5.6
138	555.3	531.3	-411.2	-387.3	23.9
139	571.4	566.6	-427.3	-422.6	4.8
140	569.3	563.5	-425.3	-419.5	5.8
141	517.5	511.1	-373.5	-367.1	6.4
142	575.6	554.8	-431.6	-410.8	20.8
143	573.2	556.5	-429.2	-412.5	16.7
144	524.3	516.5	-380.3	-372.5	7.8
145	486.0	468.1	-342.0	-324.1	17.9
146	583.5	573.4	-439.5	-429.3	10.1
147	490.1	469.9	-346.1	-325.9	20.2
148	489.9	477.7	-345.8	-333.7	12.2
149	547.2	540.0	-403.2	-396.0	7.2
150	553.9	537.4	-409.8	-393.4	16.4
151	577.5	571.1	-433.5	-427.1	6.3
152	579.4	577.2	-435.4	-433.2	2.2
153	569.0	565.1	-425.0	-421.1	3.9
154	589.0	581.2	-445.0	-437.2	7.9
155	509.4	498.9	-365.3	-354.9	10.5
156	508.3	494.1	-364.3	-350.1	14.2
157	516.8	502.3	-372.8	-358.3	14.5
158	607.5	586.6	-463.5	-442.6	20.9
159	613.7	605.1	-469.7	-461.1	8.6
160	564.4	557.6	-420.4	-413.6	6.8
161	492.8	489.6	-348.7	-345.6	3.1
162	550.3	542.3	-406.2	-398.3	7.9
163	577.4	559.2	-433.4	-415.2	18.2
164	521.1	499.4	-377.1	-355.3	21.7
165	577.0	564.4	-433.0	-420.4	12.6
166	510.2	495.2	-366.2	-351.2	15.0
167	586.7	579.3	-442.7	-435.3	7.4
168	485.5	476.1	-341.5	-332.1	9.4
169	566.5	560.2	-422.5	-416.2	6.3
170	513.4	502.5	-369.4	-358.5	10.9
171	487.9	477.5	-343.9	-333.5	10.4
172	491.0	473.0	-347.0	-329.0	18.0
173	550.3	536.9	-406.3	-392.8	13.5

174	536.9	533.2	-392.9	-389.2	3.7
175	460.9	460.6	-316.9	-316.6	0.3
176	521.8	511.7	-377.8	-367.7	10.1
177	566.1	555.2	-422.1	-411.2	10.9
178	548.1	544.4	-404.1	-400.4	3.7
179	559.3	556.8	-415.3	-412.8	2.4
180	531.7	530.8	-387.7	-386.8	0.9
AVERAGE	**542.1**	**529.4**	**-398.1**	**-385.4**	**---**
STANDARD DEVIATION	**52.6**	**54.5**	**52.6**	**54.5**	**---**

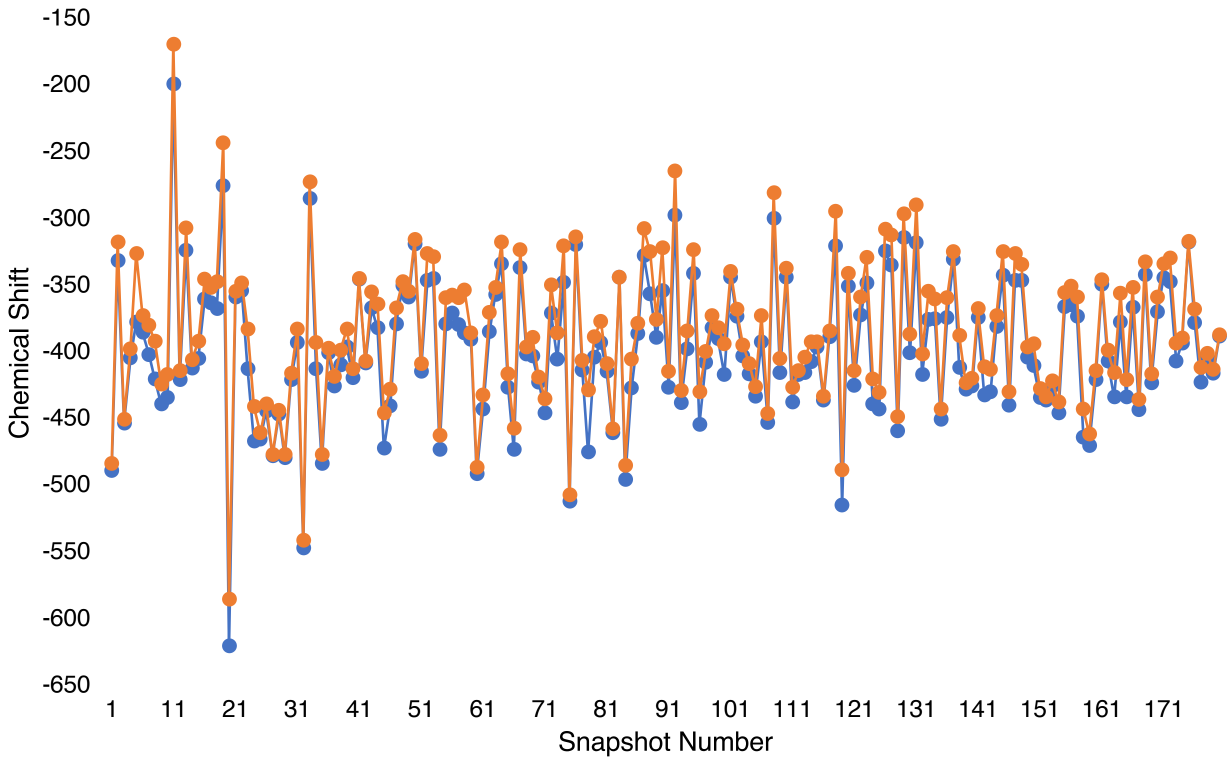

Figure S6. The plot of dynamic chemical shift values against the snapshot number. Refer to Table S6 for the data used for plotting (snapshot number and chemical shift values with and without explicit solvent molecules). Orange and blue colors show the chemical shift values with and without explicit solvent molecules, respectively.
